# Resilience and Counseling Received by Colorectal Cancer Patients During a 1-Year Follow-Up: PHASE II of a Longitudinal Study

**DOI:** 10.1097/SGA.0000000000000883

**Published:** 2025-07-29

**Authors:** Saija Pauliina Sihvola, Lauri Markus Kuosmanen, Santtu Juhani Mikkonen, Tarja Anneli Kvist

**Affiliations:** **About the authors:** Saija Pauliina Sihvola, MHSc, RN, Doctoral Researcher, Department of Nursing Science, University of Eastern Finland, Kuopio, Finland.; Lauri Markus Kuosmanen, PhD, RN, Docent, University Lecturer, Department of Nursing Science, University of Eastern Finland, Kuopio, Finland.; Santtu Juhani Mikkonen, PhD, Statistician, Department of Environmental and Biological Sciences, University of Eastern Finland, Kuopio, Finland.; Tarja Anneli Kvist, PhD, RN, Professor, Head of the Department, Department of Nursing Science, University of Eastern Finland, Kuopio, Finland.

**Keywords:** colorectal cancer, counseling, longitudinal study, patient, resilience

## Abstract

Longitudinal studies on the resilience of colorectal cancer patients and the counseling they receive are rare. This study’s purpose was threefold: to describe the resilience and received counseling of colorectal cancer patients after 1 year post surgery; the relationships between background characteristics, resilience, and counseling; and the changes that occurred in the findings. Data were collected in Finland in 2020–2023 after 1 month (phase I) and a year (phase II) of the colorectal cancer surgery using the Connor–Davidson Resilience Scale^©^ and the Counseling Quality Instrument^©^. A total of 103 participants completed phase I, 51 participants completed phase II, and 41 participants completed both phases. Phase II of the study showed that among the respondents (n = 51), resilience was low (mean 73.3) and significantly related to marital status (*p* = .007). Most (75%) evaluated counseling as good, and the evaluations were similar in both study phases (n = 103, n = 51). The lowest scores were for psychosocial support, patient-centeredness, and goal-directedness. Knowledge of the illness and its care, general health and patient attitudes, and disease treatment and self-care decreased significantly within the year (n = 41). Changes in resilience were not found (n = 41). The findings suggest that colorectal cancer patients might need more psychosocial support. Patients’ care should be carefully planned to better address their needs.

Colorectal cancer accounts for approximately 10% of all cancer cases, with about 1.9 million new cases worldwide annually (World Health Organization, 2024). Resilience is about surviving difficult situations in life. It means overcoming these situations, such as confronting serious illnesses such as cancer (Chou et al., 2023; Eicher et al., 2015; Luo et al., 2023). Luo et al. (2023) identified in their qualitative study five themes as the key components of individual resilience in adult colorectal cancer patients: seeking motivations to move forward, striving for normality, adapting and managing self, drawing on external supports, and redefining self (Luo et al., 2023). We believe that all these themes can be addressed through patient counseling. Prior studies show that the unmet psychological needs of cancer patients are strongly associated with lower resilience (Dubey et al., 2015), and patient counseling has a positive effect on colorectal cancer patients’ psychosocial and self-management skills (Faury et al., 2017).

In this study, based on Kääriäinen’s description, counseling refers to the events between the colorectal cancer patient and nursing staff during the patient’s colorectal cancer journey, taking into account the offered physical and mental support of the colorectal patients’ in their situation. In Finland, counseling is equated as a concept with advice and teaching, in which case counseling is also referred to as guidance and patient education (Kääriäinen, 2007).

## Background

Colorectal cancer patients’ counseling usually begins before a planned colorectal cancer surgery with counseling areas concerning ostomy care, sexuality, emotional condition, self-image, lifestyles, and other self-care issues, and continues after the operation with almost the same topics for the next five years (Faury et al., 2017). About one in three to one in five colorectal cancer patients receive either a temporary or permanent ostomy as part of their cancer treatment. For this reason, but not only because of that (Colorectal Cancer Alliance, 2024), patients also receive important information before the colorectal cancer surgery about the actual operation, recovery, and life after the operation. They also need to know about the potential side effects of the treatments (Koet et al., 2021).

Received knowledge, along with social and mental support, helps patients recover from illness (Koet et al., 2021; Zhang et al., 2020) and reduces the length of hospital stay (Chapman et al., 2020; Koet et al., 2021) and extra visits to health care units (Stenberg et al., 2018). Thus, counseling is an important part of cancer care (Faury et al., 2017; Pettersson et al., 2017). Its quality can be explored by asking the opinions of the health care professionals (Husebo et al., 2020); or, for example, via observation by using audio-recorded real-life situations (Pettersson et al., 2017); or by analyzing the patient education materials (Smith et al., 2014). The quality of counseling can also be explored by asking opinions from patients themselves (Lam et al., 2016; Launonen et al., 2023; Willems et al., 2016; Zebrack et al., 2014), as they are the most important sources when the issues are concerning their lives and life situations.

Prior studies show that cancer patients appreciate patient-centered and individual care (Launonen et al., 2023), interaction with staff and their peers (Appleton et al., 2018), provision of the information (Li et al., 2013), and well-planned care with clear care plans (Sprague et al., 2013). They also appreciate the professional skills of the health care staff, feeling that they are in safe hands (Appleton et al., 2018). However, cancer patients do not always get the support they need. They need more psychosocial support (Li et al., 2013; Willems et al., 2016), knowledge of potential recurrence (Willems et al., 2016), and information about the illness and its care in general (Li et al., 2013; Smith et al., 2014; Willems et al., 2016).

There are some studies concerning the causal effects of the counseling received among colorectal cancer patients (Batehup et al., 2021; Tuominen et al., 2023; Zhang et al., 2020). However, there is a lack of follow-up studies concerning resilience among adult cancer patients (Cosco et al., 2017; Eicher et al., 2015), particularly with quantitative measures among colorectal cancer patients (Sihvola et al., 2022; Sihvola, Kiwanuka, & Kvist, 2023). Previous studies have shown that after years of cancer care, patients may still have several problems caused by the cancer and its treatments (Lam & Jones, 2020; Nikoletti et al., 2008), as well as psychosocial needs that have not been addressed (Willems et al., 2016; Zebrack et al., 2014). Thus, we explored colorectal cancer patients who had their surgery 1 year ago, their evaluations of resilience and the received counseling, and the changes in the findings.

## Materials and Methods

### Purpose of the Study

The study’s purpose was threefold: to describe the resilience and received counseling of colorectal cancer patients after 1 year of the surgery; the relationships between background characteristics, resilience, and counseling; and the changes that occurred in the findings.

The study questions were as follows: (1) How do the patients evaluate their resilience 1 year after the surgery? (2) Which background characteristics are related to resilience 1 year after the surgery? (3) How do the patients evaluate the received counseling 1 year after the surgery? (4) What are the possible background characteristics that are related to the received counseling 1 year after the surgery? (5) How are counseling and resilience related to each other 1 year after the surgery? (6) What are the differences between two measurement times: 1 month and 1 year after the surgery? (7) How have the patients’ evaluations of resilience and counseling changed within the year?

### Study Design

This research employed a descriptive longitudinal study design with two questionnaire phases.

### Participants

About 3,800 new colorectal cancer cases are diagnosed in Finland every year (Finnish Cancer Registry, 2021). Thus, based on power analysis (annual new cases in Finland, N = 3,800, confidence level 95%), the target sample was n = 349. However, the study samples consisted of voluntary participants in phase I, about 1 month after their colorectal cancer surgery, and in phase II, about 1 year after their surgery. Eligibility criteria for participants included being 18 years of age or older, having completed scheduled colorectal cancer surgery, and having received care in the Finnish university hospital. The exclusion criteria were: memory disorder or another neurological disease that made it difficult for the patient to think and express themselves, or the phase of the recovery process in which the patient cannot familiarize themselves with the information provided about the study and make a decision about participating in it.

### Data Collection

Data were collected 1 month after the surgery and again 1 year after the surgery in Finland between autumn 2020 and autumn 2023. The participants of this study were recruited during their hospital stay for colorectal cancer surgery or at their first outpatient visit 2 to 4 weeks post-surgery with the assistance of nurses who worked in the Finnish university hospitals. All nurses received both verbal and written instructions on how to recruit the potential participants. The potential participants received a verbal and written presentation of the research study. The informational materials also included the contact information of the researchers, so the potential participants had the opportunity to directly contact the researchers through email or by phone.

After the provided information about the study, patients were recruited as voluntary participants. When the participants had received the information about the research and expressed their interest in participating in the longitudinal study, they were asked to give their informed consent by signing a consent form. After consent, the participants received the first questionnaire. The answers were mailed to the researchers in a pre-paid envelope about 1 month after the colorectal cancer surgery (phase I). Each consent form and questionnaire included a code combining the participants’ collected information. The codes enabled to send in phase II the second questionnaire directly for the home addresses of the consented participants one year after the surgery. Similarly as in phase I, participants send their answers directly to the researcher, to her university address.

### Ethical Approval and Research Permissions

The study had approval from the ethics committee of the health care district of Northern Savo (registry number 1850/13.02.00/2019) and obtained research permissions from the participating organizations: Helsinki University Hospital (HUS/115/2020), Kuopio University Hospital (50H0/42), Oulu University Hospital (210/2020), Turku University Hospital (T03/015/20), and Tampere University Hospital (R21014). Permission to use the Connor–Davidson Resilience Scale^©^ and information concerning the data handling were requested from one of the developers of the scale, Emeritus Professor Jonathan Davidson, and for the Counseling Quality Instrument^©^ (CQI) from the instrument developer Professor Maria Kääriäinen. The study was conducted in accordance with the Code of Ethics of the Declaration of Helsinki (World Medical Association, 2023).

### Measures, Validity, and Reliability

The scales that were used in this longitudinal study were Connor–Davidson Resilience Scale^©^ (CD-RISC-25) and the CQI. The CD-RISC-25 consisted of 25 items, and answers were given on a five-point Likert scale ranging from 0 to 4 (0 = not true at all, 4 = true nearly all of the time). The total score ranges from 0 to 100, with high scores reflecting better resilience. Participants are asked to respond to which choice best describes their situation during the past month (Connor & Davidson, 2003). The scale’s Cronbach’s alpha has previously been reported to be between 0.85 and 0.89 (Ҫakir et al., 2020; Connor & Davidson, 2003; Zhang et al., 2020). In this study phase, it was 0.88.

The CD-RISC-25 covers different aspects of resilience: hardiness, coping, adaptability/flexibility, meaningfulness/purpose, optimism, regulation of emotion and cognition, and self-efficacy. The total mean scores of resilience in the general population have been categorized as follows: 0–73 as lowest; 74–82 as the second quartile; 83–90, third quartile; and the highest score quartile, 91–100 (Connor & Davidson, 2020).

Counseling quality was measured using the CQI^©^. The CQI includes 125 items that measure counseling quality on a five-point Likert scale ranging from 1 to 5 (1 = strong disagreement, 5 = strong agreement), with 1.00–2.49 reflecting poor, 2.50–3.49 reflecting satisfactory, and 3.50–5.00 reflecting good patient counseling (Kääriäinen, 2007). Participants were asked to respond to which choice best describes the received counseling to date. CQI’s Cronbach’s alpha value has previously been reported to be between 0.78 and 0.97 (Kääriäinen, 2007; Rajala et al., 2018). In this study phase, it was 0.79.

The CQI covers four main subareas: (1) content of counseling, (2) implementation of counseling, (3) benefits of counseling, and (4) counseling resources. The “content” aspect evaluates whether the counseling provided was sufficient, such as whether patients received adequate guidance on nutrition post-operation. The “implementation” aspect examines, for example, whether the counseling was patient-centered and goal-oriented and whether the interaction was good between a patient and a health care professional. The “benefits” aspect assesses the utility of counseling; for instance, its role in enhancing patients’ confidence in their future or overall well-being. “Resources” includes items that discover, for example, whether the health care professional had enough time for the counseling and whether the counseling methods used were appropriate (Kääriäinen, 2007). The CQI also includes a question about total counseling quality in which respondents are asked to rate a single item on a five-point scale ranging from 1 (poor) to 5 (excellent). In addition, it includes two questions about total counseling quality before and after surgery, which respondents are asked to rate on a five-point scale ranging from 1 (strong disagreement) to 5 (strong agreement) (Kääriäinen, 2007).

The content and benefits of the counseling were specially adapted for this study with permission and introductions from the instrument developer, meaning 22 added questions to the CQI subareas: content of counseling and benefits of counseling (Kääriäinen, 2023). The added questions included content related to the counseling of colon cancer patients, topics such as cancer prognosis and recurrence risk, topics related to stoma care, and social benefits and support groups. Questions related to the benefits of counseling included questions about, for example, whether the counseling had helped in lifestyle change and whether the counseling had promoted the patient’s confidence in the future. A colorectal cancer patient, ostomy nurse, and cancer physician participated in planning the questionnaire concerning the background questions and the additional questions in the CQI. In addition, three colorectal cancer patients tested the entire questionnaire before the study by answering the questions and evaluating the suitability of the questionnaire.

After testing the questionnaire and before the study phase I, the research group approved it, and the questionnaire remained similar for both study phases as it was before the tests. Researchers sought to avoid placing any pressure on patients to participate in the study. Therefore, the ethical decision was made to not send reminders about answering the survey. Patients had the right to not answer the survey, even if they had previously consented to participate in the study.

The background characteristics (n = 7) were: *gender* (1 = female, 2 = male), *age* (phase I: 1 = 34–65 years, 2 = 66–84 years; phase II: 1 = 46–65 years, 2 = 66–83 years), *work status* (1 = full or part-time job, 2 = retired, or otherwise unemployed), *marital status* (phase I: 1 = unmarried, 2 = live together/married, 3 = divorced, 4 = widowed; phase II: 1 = unmarried, divorced, or widowed; 2 = live together/married), *smoking status* (1 = non-users, 2 = users of cigarettes or snuff, or electronic cigarettes), *alcohol consumption* (1 = abstainers, 2 = consuming 2–4 times a month, 3 = consuming four times a week or more), *and presence of an ostomy* (1 = yes, 2 = no).

### Data Analysis

Data were collected using descriptive statistics (frequencies, percentages, mean scores, and standard deviations). The mean resilience (25 items) was calculated at the total scale level, and the possible score range was 0–100. The mean item scores of *counseling quality* were summed at the subscale level, while the possible mean scores ranged from 1 to 5. Within the content of the *counseling quality* “subareas”, three sum variables were constructed: (1) “knowledge of the illness and its care” (12 items); (2) “care and condition after the surgery” (12 items); and (3) “psychosocial support” (6 items). From the *implementation of counseling* subarea, three sum variables were compiled: (1) “interaction of counseling” (14 items); (2) “patient-centered counseling” (9 items); and (3) “goal-directed counseling” (12 items). In the *benefits of counseling* subarea, two sum variables were delineated: (1) “general health and patient attitudes” (7 items) and (2) “disease treatment and self-care” (14 items). A single sum variable consolidated all variables related to the *counseling resources* (10 items) (Kääriäinen, 2023). *Counseling quality* scores were categorized as follows: 1.00–2.49 indicated “poor” patient counseling, 2.50–3.49 indicated “satisfactory counseling, and 3.50–5.00 signified”good“patient counseling” (Kääriäinen, 2007).

The significance of relationships between background variables (age, gender, work status, marital status, smoking status, alcohol consumption, and presence of ostomy) and the total resilience score, as well as between background variables and sum variables of the counseling subareas, were examined as skewed distributions with non-parametric tests (Mann–Whitney *U* test and Kruskal–Wallis test) and as symmetrical with parametric tests (independent samples *T*-tests and analysis of variance).

The difference between the first and second phases was measured with descriptive statistics (n = 103, n = 51), and the changes that occurred were assessed with paired *t*-test (n = 41, n = 41). As such, when making comparisons, we used the full samples (n = 103, n = 51) and samples that included the same participants in phases I and II (n = 41, n = 41). Spearman’s correlation analysis was used to test the relationships between resilience and the sum variables of the counseling subareas.

The internal consistency of CD-RISC-25 (Connor & Davidson, 2003) and the CQI (Kääriäinen, 2007) was examined through Cronbachʼs alpha values. Data were analyzed using IBM SPSS Statistics for Windows, Version 27 (IBM Corp, Armonk, New York). A statistically significant *p*-value was set as ≤.05. This study adhered to the Strengthening the Reporting of Observational Studies in Epidemiology (STROBE) statement guidelines for reporting observational studies.

## Results

### Respondent Characteristics

The study samples consisted of 103 voluntary participants in phase I, about 1 month after colorectal cancer surgery, and 51 participants in phase II, about 1 year after their surgery. Of the 51 participants, 10 did not answer the first phase but answered the second questionnaire, and therefore, there were a total of 41 participants who answered both study phases. The participants had completed scheduled colorectal cancer surgery and had received care in the Finnish university hospital.

**Phase I:** A total of 103 participants completed the phase I questionnaire about 1 month after their colorectal cancer surgery. Most of them were males (62%), and most of them had an ostomy (79%). Participants were aged between 34 and 84 years, and their mean age was 66 years. Most were non-smokers (90%), retired, or otherwise unemployed (75%), and they were living together with someone or were married (72%). Of the participants, 18% were not using alcohol at all, 29% used it once a month or less frequently, 41% used it 2–4 times a month, and 12% used it four times a week or more frequently.

**Phase II**: A total of 51 participants (out of 103 phase I participants) provided data in phase II, about 1 year after the colorectal cancer surgery. Most of the respondents were males (76%), and 37% had an ostomy. Participants were aged between 46 and 83 years, and their mean age was 68 years. Most were non-smokers (94%), retired, or otherwise unemployed (78%), and they were living together with someone or were married (78%). Of the participants, 20% were not using alcohol at all, 29% used it once a month or less frequently, 43% used it 2–4 times a month, and 8% used it four times a week or more frequently.

**Phase I and II**: A total of 41 participants answered both: phase I and phase II. Most of them were males (72%). In phase I, the majority had an ostomy (90%), while in phase II, the majority did not have it (61%). In phases I and II, participants were aged between 34 and 84 years, and their mean age was 68 years. Most of the participants in phases I and II (93%) were non-smokers and living together with someone or married (78%). In phases I and II, most were retired, or otherwise unemployed (76% and 78%, respectively). Of the participants in phases I and II, 20% were not using alcohol at all, and 29% used it once a month or less frequently. In phase I, 44% used alcohol 2–4 times a month, while this kind of consumption decreased within the year, and in phase II, the same percentage was 42%. In phase I, 7% used alcohol four times a week or more frequently, while this kind of alcohol consumption within the year increased, and in phase II, the same percentage was 10%.

### Resilience and Related Background Characteristics 1 Year After the Surgery

A total CD-RISC score among the colorectal cancer patients (n = 51) in phase II of this longitudinal study was 73.3, SD 13.3, ranging between 44 and 95 (scale 0–100) with a symmetrical distribution. The independent samples *T*-test revealed that gender, age, work status, smoking status, or presence of an ostomy were not statistically significant factors affecting resilience findings. Analysis of variance confirmed that alcohol consumption also was not statistically significant.

However, independent samples *T*-test revealed that marital status was a statistically significant factor related to resilience (*t* = −3.049, *df* 16.65, *p* = .007), meaning that respondents who were unmarried, divorced, or widowed evaluated resilience lower than participants who were living together with someone or were married (mean 63.1, SD 11.9 vs. mean 75.6, SD 12.5, respectively).

### Received Counseling and Related Background Characteristics 1 Year After the Surgery

Most (75%) of the total 51 study participants evaluated that *total counseling quality* had been “good” or “excellent”, while 15% evaluated that it had been at a “satisfactory” level, and a minority evaluated it as “poor” or “below” average (10%). Most of the respondents evaluated that counseling had been “;good,” both before (86%) and after the surgery (92%). From the subarea content of the *quality counseling* quality, participants rated the highest for *Knowledge of the illness and its care* (mean 3.9, SD 0.88), while the lowest rating was for *psychosocial support* (mean 3.2, SD 1.08), which was at the “satisfactory” level.

According to participants, counseling was implemented well if considering its *interaction*, which was evaluated as the highest in the subarea of *implementation of the counseling* (mean 3.7, SD 1.25). Counseling was beneficial if considering both of “general health and patient attitudes” and “disease treatment and self-care”, which were evaluated as “good” in the subarea of *benefits of the counseling* (mean 3.9, SD 1.08 and mean 3.9, SD 1.16, respectively). Moreover, most of the respondents agreed “partly” or “fully” (64%) that counseling had enhanced their trust in the future (mean 3.9, SD 1.18). The *counseling resources* were evaluated as “good” (mean 4.0, SD 0.97).

However, the independent samples *T*-test and the analysis of variance revealed that there was no statistically significant relationship between respondents’ background characteristics (gender, age, work status, smoking status, presence of an ostomy, or alcohol consumption) and “goal-directedness, patient-centeredness, psychosocial support”, or “knowledge of the illness and its care”. Moreover, Mann–Whitney *U* test and Kruskal–Wallis test revealed that there was not a statistically significant relationship between background characteristics and “interaction of the counseling, resources of the counseling, care and condition after the surgery, disease treatment and self-care”, or “general health and patient attitudes”.

### The Relationships Between Resilience and Counseling Quality 1 Year After the Surgery

Resilience was statistically significantly related to care and condition after the surgery (rho = 0.287), which belongs in the subarea content of *counseling quality*. This kind of relationship was not found for the other summed variables of the counseling subareas.

### Comparisons of Resilience and Received Counseling at the Questionnaire Phase I and II

The total mean CD-RISC score among the colorectal cancer patients (n = 103) 1 month after the surgery was 70.7, SD 15.6, and 1 year after the surgery (n = 51), it was 73.3, SD 13.3. Comparisons between two measurement times (n = 103, n = 51) concerning resilience with background characteristics are presented in Table 1. Frequencies and percentiles of the two measurement times of the longitudinal study (phase I and II) concerning the received counseling (n = 103, n = 51) are presented in Table 2.

**TABLE 1.** Comparisons of Resilience Between Two Phases (Phase I N = 103, Phase II N = 51)

Background Characteristics	Phase I		Phase II	
		Resilience		Resilience
	Number (%)	Mean, SD	Number (%)	Mean, SD
Gender				
Female	39 (37.9)	74.1 ± 16.4	12 (24.0)	72.0 ± 17.1
Male	64 (62.1)	69.0 ± 15.1	38 (76.0)	73.0 ± 12.8
*Missing*	0	0	1	0
Age				
Phase I 34–65 years, Phase II 46–65 years	43 (42.6)	73.4 ± 12.9	18 (37.5)	76.1 ± 13.1
Phase I 66–84 years, Phase II 66–83 years	58 (57.4)	69.0 ± 17.1	30 (62.5)	70.9 ± 13.9
*Missing*	2	0	3	0
Work status				
Full- or part-time job	26 (25.5)	69.6 ± 12.7	11 (22.0)	73.0 ± 9.08
Retired or unemployed	76 (74.5)	70.8 ± 16.5	39 (78.0)	72.8 ± 14.53
*Missing*	1	0	1	0
Marital status				
Unmarried, divorced, widowed	29 (28.4)	71.2 ± 18.3	11 (21.6)	63.1 ± 11.86
Live together/married	73 (71.6)	70.3 ± 14.5	40 (78.4)	75.6 ± 12.51
*Missing*	1	0	0	0
Smoking status				
Never use any	91 (90.1)	70.7 ± 15.6	48 (94.1)	72.5 ± 13.4
Using cigarettes or snuff or cigars or electronic cigarettes	10 (9.9)	72.9 ± 17.6	3 (5.9)	78.3 ± 12.2
*Missing*	2	0	0	0
Alcohol consumption				
Never use any	19 (18.4)	73.6 ± 20.6	10 (19.6)	68.5 ± 17.1
About once a month or less frequently	30 (29.1)	71.8 ± 12.8	15 (29.4)	74.2 ± 12.1
2–4 times a month	42 (40.8)	71.7 ± 15.9	22 (43.1)	75.6 ± 12.3
Four times a week or more frequently	12 (11.7)	60.0 ± 9.8	4 (7.8)	62.5 ± 17.1
*Missing*	0	0	0	0
Presence of an ostomy				
Yes	81 (79)	72.9 ± 15.0	19 (37.3)	69.3 ± 14.0
No	22 (21)	62.9 ± 16.1	32 (62.7)	74.7 ± 13.4
*Missing*	0	0	0	0

**TABLE 2.** Comparisons Between Two Measurement Times Concerning the Received Counseling (Phase I N = 103, Phase II N = 51)

Subarea of the Counseling	Phase I					Phase II				
	Good n (%)	Satisfactory n (%)	Poor n (%)	Mean, SD	Missing n	Good n (%)	Satisfactory n (%)	Poor n (%)	Mean, SD	Missing n
Content of counseling										
Knowledge of the illness and its care	81 (79.4)	12 (11.8)	9 (8.8)	3.9 ± 0.86	1	38 (74.5)	8 (15.7)	5 (9.8)	3.9 ± 0.88	0
Care and condition after the surgery	70 (68.6)	19 (18.6)	13 (12.7)	3.7 ± 0.87	1	35 (68.6)	12 (23.5)	4 (7.8)	3.8 ± 0.92	0
Psychosocial support	42 (41.2)	32 (31.4)	28 (27.5)	3.1 ± 1.09	1	23 (46.0)	16 (32.0)	11 (22.0)	3.2 ± 1.08	1
Implementation of counseling										
Patient-centeredness	34 (41.0)	26 (31.3)	23 (27.7)	3.1 ± 1.10	20	17 (41.5)	14 (34.1)	10 (24.4)	3.2 ± 1.21	10
Interaction	58 (69,9)	16 (19.3)	9 (10.8)	3.8 ± 1.10	20	27 (64.3)	8 (19.0)	7 (16.7)	3.7 ± 1.25	9
Goal-directedness	37 (44.0)	25 (29.8)	22 (26.2)	3.1 ± 1.15	19	16 (39.0)	14 (34.1)	11 (26.8)	3.2 ± 1.20	10
Benefits of counseling										
General health and patient attitudes	56 (70.9)	16 (20.3)	7 (8.9)	3.8 ± 1.05	24	27 (67.5)	10 (25.0)	3 (7.5)	3.9 ± 1.08	11
Disease treatment and self-care	51 (64.6)	20 (25.3)	8 (10.1)	3.8 ± 1.09	24	29 (70.7)	7 (17.1)	5 (12.2)	3.9 ± 1.16	10
Counseling resources	73 (85.9)	8 (9.4)	4 (4.7)	4.2 ± 0.84	18	30 (73.2)	9 (22.0)	2 (4.9)	4.0 ± 0.97	10

3.50–5.00 "good"; 2.50–3.49 "satisfactory", and 1.00-2.49 "poor".

### Changes in Resilience and Received Counseling Between the First and Second Phase

Paired sample*t*-test revealed that there were no positive changes in resilience and received counseling. In the subarea content of the *counseling quality*“knowledge of the illness and its care, general health and patient attitudes”, and “disease treatment and self-care” decreased within the year among the respondents (n = 41) who answered both phases (mean 4.09, SD 0.65 vs. mean 3.83, SD 0.92, *p* = .025; mean 3.62, SD 1.12 vs. mean 2.56, SD 0.70, *p* < .001; mean 3.86, SD 1.07 vs. mean 2.58, SD 0.64, *p* < .001, respectively). Evaluations of resilience or the other summed variables in the *counseling quality* subareas showed no statistically significant differences within the year (Table 3).

**TABLE 3.** Changes of the Experiences in Resilience and Received Counseling (N = 41)

Variables	Phase I	Phase II	
	Mean, SD	Mean, SD	Paired Samples *T*–Test, *p*-Value
Resilience	71.22 ± 12.90	71.73 ± 13.00	.859
Content of counseling (quality)			
Knowledge of the illness and its care	4.09 ± 0.65	3.83 ± 0.92	.025^a^
Care and condition after the surgery	3.81 ± 0.80	3.80 ± 0.97	.907
Psychosocial support	3.23 ± 1.12	3.22 ± 1.09	.952
Implementation of counseling			
Patient-centeredness	3.16 ± 1.13	3.10 ± 1.24	.702
Interaction	3.63 ± 1.19	3.65 ± 1.28	.936
Goal-directedness	3.13 ± 1.15	3.09 ± 1.24	.752
Benefits of counseling			
General health and patient attitudes	3.62 ± 1.12	2.56 ± 0.70	<.001*
Disease treatment and self-care	3.86 ± 1.07	2.58 ± 0.64	<.001*
Counseling resources	4.18 ± 0.87	3.97 ± 0.92	.205

^a^ *p*-value ≤ .05.

Evaluations of the counseling:1.00–2.49 “poor”, 2.50–3.49 “satisfactory”, 3.50–5.00 “good”.

## Discussion

The total CD-RISC mean score among the colorectal cancer patients (n = 51) in phase II of this longitudinal study was a little better than in phase I (n = 103) (mean 73.3 vs. mean 70.7, respectively). Although the change in resilience was not statistically significant, this finding supports the definition that resilience is a process in which a person (Chou et al., 2023; Eicher et al., 2015; Luo et al., 2023) strives for normality (Luo et al., 2023). However, the respondents’ resilience level in this longitudinal study phase I and II was at the lowest level when comparing it to the general population sample (Connor & Davidson, 2020) or to the Turkish sample of colorectal cancer patients (mean 78.68) in their early phase of cancer (Ҫakir et al., 2020). It is not know if resilience is normally this low among the Finnish patients, or if the low resilience was caused by the COVID-19 pandemic during the data collection.

One year after the surgery, marital status was the only statistically significant factor of the background characteristics that were related to resilience (*p* = .007), meaning that respondents who were unmarried, divorced, or widowed evaluated resilience lower than participants who were living together with someone or were married. This finding is congruent (i.e., quality) with previous studies, which have shown that resilience is associated with marital status (Bradley & Hojjat, 2017; Timalsina et al., 2021), and in fact, resilience is one of the main predictors of marital satisfaction (Bradley & Hojjat, 2017). In this study, the relationship between resilience and other background characteristics was not found despite some variations in CD-RISC mean scores.

Resilience was statistically significantly related to the content of counseling (i.e., quality), a summed variable of “care and condition 1 year after the surgery” (rho = 0.287), which supports the general purpose of the counseling, which is to enhance a patient’s knowledge of the illness and its care (Chiu et al., 2017). This finding also supports the fact that resilience enhances the adjustment to a patient’s condition (Tirgari et al., 2023).

Most of the study participants (75%) evaluated total *counseling quality* as “;good” or “excellent”, as well as “good” before (86%) and after the surgery (92%). Moreover, frequencies and percentiles of the two measurement times (n = 103 and n = 51) showed that the evaluations concerning the received counseling were very similar, mainly “good”, in both study phases of this longitudinal study. These findings are positive because well-planned and quality counseling enhances recovery after surgery (Forsmo et al., 2016).

In this longitudinal study phase II, *counseling quality* was sufficient when considering its subareas “knowledge of the illness and its care” and “care and condition after the surgery”, good when considering “counseling resources” and “interaction of the counseling”. Counseling was beneficial if considering “general health and patient attitudes” and “disease treatment and self-care,” which were both evaluated as “good”. Moreover, most of the respondents agreed “partly” or “fully” (64%) that counseling had enhanced their trust in the future, which is an important aspect of resilience (Connor & Davidson, 2003). However, the lowest ratings were given to “psychosocial support”, “patient-centeredness”, and “goal-directedness”, which were evaluated similarly in phase I.

These findings underline the importance of comprehensive counseling, where clear goals set for counseling can assist patients on their cancer journey and better address patients’ needs (Sihvola, Kiwanuka, & Kvist, 2023). Moreover, the important finding of this longitudinal study was that evaluations of the “knowledge of the illness and its care” (content), “general health and patient attitudes” (benefits), and “disease treatment and self-care” (benefits) decreased within the year among the respondents who answered both phases (n = 41), reflecting a possible need to provide more attention to later phases of cancer care (Willems et al., 2016) and long-term goals for the counseling. In addition, although there was a little better resilience when comparing the results between phases I and II, the actual changes in resilience were not found (n = 41).

The findings of this study suggest that counseling still needs to be developed. More attention should be given to the provision of psychosocial support (Willems et al., 2016; Zebrack et al., 2014). In addition, health care professionals should be aware if patients have close and secure relationships to support them in their cancer journey. Previous research by Zeilani et al. (2022) showed that these kinds of relationships help patients to survive cancer, assist them in decisions related to their treatment, and overcome possible fears caused by cancer. Furthermore, this study highlights the importance of planned care. Patient counseling is not always planned with clear goals (Rajala et al., 2018), which would enable health care professionals to better address patients’ needs, assist them to adequately cope with their concerns, and support them in their recovery process (Sihvola, Kiwanuka, & Kvist, 2023).

## Practice Implications

Health care professionals should be aware if patients have close and secure relationships to support them in their cancer journey because if these relationships are lacking, patients might need more support from health care professionals. The evaluations of the knowledge of the illness and its care, general health and patient attitudes, and disease treatment and self-care decreased significantly within the year, which support the aspect that colorectal cancer patients might need more support after their surgery. Furthermore, health care professionals should pay more attention to the provision of psychosocial support and planned care, which enable them to better address patients’ needs.

## Strengths and Limitations

The strength of this study is that it addressed rarely studied resilience in this quantitative follow-up study among colorectal cancer patients, as well as comprehensively their perceptions of the received counseling. In addition, these data were valuable, although it was gathered during the COVID-19 pandemic, because it can also inform something about respondents’ situations and cancer care during the global crisis. However, it was very difficult to get these data during that crisis, taking about 3 years to gather it. This is understandable because the study participants were recruited with the assistance of nurses, who were certainly challenged during the pandemic (Sihvola, Nurmeksela et al., 2023b) along with the global nurse shortage (International Council of Nurses, 2023).

One limitation of this study is the missing responses in some parts of the questionnaire in both phases of the study. Furthermore, there were not equal participant numbers who answered both, in phases I and II. All these made it difficult to compare changes among the dependent variables. However, the missing responses were reported, comparisons were made for both phases (n = 103 and n = 51), and changes in experiences and evaluations were explored among those who answered both phases (n = 41).

In this study, the samples were based on power calculation. However, the actual samples were small. The small sample sizes in both arms of the longitudinal study make it difficult to generalize the findings. However, his study presented both positive and negative findings, as well as changes in the experiences among colorectal cancer patients, about a topic that is rarely studied in quantitative follow-up studies, especially in Europe (Sihvola et al., 2022; Sihvola, Kiwanuka, & Kvist, 2023).

## Conclusion

Resilience of Finnish colorectal cancer patients was at a low level and remained low within the 1-year follow-up. This warrants more research to address this topic because there is a lack of knowledge to determine if resilience is normally this low among these Finnish patients, or was the low resilience caused by the COVID-19 pandemic during the data collection. In this study, the marital status of the patients was significantly related to resilience, which highlights the importance of external resources in patient’s cancer journey. A positive finding in this study was that the overall evaluations of the counseling were mostly good. However, decreased knowledge of the illness and its care suggests that patients have still information and support needs that should be addressed because they can be beneficial for patients’ self-care and enhance their general health. More attention should be given to the provision of psychosocial support and setting clear goals for the different aspects of counseling sessions and care phases.
